# Bacterial communities of Antarctic lichens explored by gDNA and cDNA 16S rRNA gene amplicon sequencing

**DOI:** 10.1093/femsec/fiad015

**Published:** 2023-02-07

**Authors:** Aleksandra Woltyńska, Jan Gawor, Maria A Olech, Dorota Górniak, Jakub Grzesiak

**Affiliations:** Institute of Biochemistry and Biophysics, Polish Academy of Sciences, Pawińskiego 5A, 02-106 Warszawa, Poland; Institute of Biochemistry and Biophysics, Polish Academy of Sciences, Pawińskiego 5A, 02-106 Warszawa, Poland; Institute of Botany, Jagiellonian University, Gronostajowa 3, 30-387 Krakow, Poland; Department of Microbiology and Mycology, University of Warmia and Mazury in Olsztyn, Oczapowskiego 1a, 10-719 Olsztyn, Poland; Institute of Biochemistry and Biophysics, Polish Academy of Sciences, Pawińskiego 5A, 02-106 Warszawa, Poland

**Keywords:** bacterial diversity, lichen holobiont, operational taxonomic units, polar regions, targeted metatranscriptomics, Usnea

## Abstract

Recently, lichens came once more into the scientific spotlight due to their unique relations with prokaryotes. Several temperate region lichen species have been thoroughly explored in this regard yet, the information on Antarctic lichens and their associated bacteriobiomes is somewhat lacking. In this paper, we assessed the phylogenetic structure of the whole and active fractions of bacterial communities housed by Antarctic lichens growing in different environmental conditions by targeted 16S rRNA gene amplicon sequencing. Bacterial communities associated with lichens procured from a nitrogen enriched site were very distinct from the communities isolated from lichens of a nitrogen depleted site. The former were characterized by substantial contributions of Bacteroidetes phylum members and the elusive Armatimonadetes. At the nutrient-poor site the lichen-associated bacteriobiome structure was unique for each lichen species, with chlorolichens being occupied largely by Proteobacteria. Lichen species with a pronounced discrepancy in diversity between the whole and active fractions of their bacterial communities had the widest ecological amplitude, hinting that the nonactive part of the community is a reservoir of latent stress coping mechanisms. This is the first investigation to make use of targeted metatranscriptomics to infer the bacterial biodiversity in Antarctic lichens.

## Teaser

Habitat location and species-specific features shape the bacterial community structure in Antarctic lichens.

## Introduction

Lichens, or rather lichenized fungi (the fungus being the main morphology- and classification-determining component), are one of the most nonconspicuous, yet relatively productive elements of terrestrial habitats worldwide (Asplund and Wardle 2017). They are primarily defined as a self-sustaining, mutualistic symbiosis between an Ascomycete fungus (mycobiont) and a photobiont, which can either be a green algae (chlorolichens), a cyanobacterium (cyanolichens), or both (tripartite lichens). These organisms, representing different kingdoms, associate into characteristic and species-specific structures, called the vegetative thalli. Both components are highly codependent, as the mycobiont provides a microhabitat, as well as water and mineral salts, whereas the photobiont supplies photosynthetically fixed carbon in the form of sugars and sugar alcohols (Aschenbrenner et al. 2016, Honegger 1991). Lichenization seems to be a very successful survival strategy among fungi, as 18% of the recognized species are capable of this kind of symbiosis, which can be traced back at least 415 million years, to the early Devonian era (Hawksworth 2001, Honegger et al. 2013). Lichens display an epiphytic or epilithic lifestyle, growing on the barks of trees, on bare rock, or compacted soil. They are estimated to cover up to 8% of the Earth’s terrestrial landscape (Ahmadjian 1995).

The redefinition of what makes up a lichen has been established only recently, in large part due to the emergence of meta-“omic” technologies (Cernava et al. 2017, Grimm et al. 2021). By the updated definition, presented by Hawksworth and Grube (2020) lichens are “a self-sustaining ecosystem formed by the interaction of an exhabitant fungus and an extracellular arrangement of one or more photosynthetic partners and an indeterminate number of other microscopic organisms,” amending the classic definition with the existence of a diverse, intrathallic consortium of microscopic organisms, consisting of nonphotosynthetic bacteria and archaea, accessory fungi and algae (Grimm et al. 2021). Several researchers indicate that this multiorganismal consortium likely dwells within the lichen thallus, surrounded by extracellular substances secreted by more than one component of this consortium. This complex biofilm layer composed of polysaccharides, such as glucans and mannans, can be considered an extracellular interaction matrix, providing a medium, in which an exchange of nutrients and signaling molecules takes place between different partners of the symbiosis (Tuovinen et al. 2019, Spribille et al. 2020). By employing culture-independent methods, a substantial part of this newly discovered lichen-associated community was identified as heterotrophic bacteria (Pankratov et al. 2017). Intra- and extrathallic activity of these bacteria may be of pivotal importance for lichen growth and survival. Metaproteomic and culture-based studies showed lichen bacteriomes’ potential for dinitrogen fixation and rock weathering—features involved in biogenic element acquisition, consequently making them available to lichen partners (Liba et al. 2006, Grube et al. 2015, Eymann et al. 2017). Lytic activities like chitynolysis, proteolysis, and glucanolysis also have been noticed, which presumably aid in recycling of old thalli parts (Sigurbjörnsdóttir et al. 2016). Lichen-associated bacteria are also responsible for vitamin and cofactor supply to the thallus, as well as furthering the growth and development of the lichen through phytohormone production (Grube and Berg 2009, Noh et al. 2021). Resistance to many abiotic factors, such as low temperatures, desiccation, and oxidative stress (Eymann et al. 2017, Grimm et al. 2021) may also be mediated by lichenophilic bacteria. Additionally, through the production of secondary metabolites, some with antagonistic effects on other microorganisms, the bacteriome poses as a major factor in the formation of the lichen species-specific microbiome (Grube et al. 2019).

Despite there being over 20 000 species described, lichens are often overshadowed in their natural habitats by the highly diverse vascular plants (Cornelissen et al. 2007, Grimm et al. 2021). However, in high latitude regions, like Antarctica, the opposite is true (Armstrong 2017). With over 400 species detected, lichens vastly outnumber resident plants in regard to biodiversity, since Antarctica houses only two species of flowering plants—*Deschampsia antarctica* and *Colobanthus quitensis* (Alberdi et al. 2002). Despite being very successful in extreme and cold habitats, lichen distribution in the Antarctic region, similarly as in the remaining parts of the terrestrial biosphere, is governed by several external factors. Due to the lack of roots, an epidermis nor a cuticle, the lichens’ metabolic activity is highly influenced by the local microclimate, geological substrate, as well as hydrological regime, among others (Armstrong 2015). However, the main factor which contributes to the formation of specific lichen communities in Antarctica, is the presence or the absence of a peculiar form of nitrogen enrichment (Bokhorst et al. 2019). This enrichment, experienced mainly at old and contemporary penguin nesting sites, is caused by the ammonification of uric acid from bird guano. Released ammonia vapors create a phenomenon known as an “ammonia shadow,” which fertilizes rocks and ground surfaces near large penguin colonies (Tatur et al. 1997, Grzesiak et al. 2020). In such conditions, ornithocoprophilous communities that are highly nitrophilic and cannot be found elsewhere in the otherwise barren Antarctic landscape, develop. Nitrophobic communities also occur in the region, albeit can be found growing exclusively where nutrient inflow is minor and rather indirect, therefore, high nitrogen concentrations can be avoided, primarily on rocky substrates inland, at higher altitudes. Both of these communities are composed of, to a certain extent, endemic, specialist lichen species (obligatory nitrophiles or nitrophobes), while also containing nitrogen-tolerant generalist lichen species, which grow regardless of nitrogen compound concentrations (Olech 2004, Johansson et al. 2011).

With the latest scientific literature being increasingly amended with reports on lichen-associated microbiomes, the issue of how the bacterial communities’ phylogenetic structure changes depending on nitrogen content preference of its host lichen has never been investigated. Thus, with the use of up-to-date, high-throughput technologies, we assessed the phylogenetic structure of the whole and active fraction of bacterial communities of Antarctic lichens growing in different environmental conditions. Our hypothesis states that nitrogen compound concentrations in the lichens preferred habitat exerts a greater influence on its associated bacteriobiome than the specific features of the host. We also hypothesize that lichens able to host a greater diversity of bacteria also have a wider niche/habitat range.

In order to prove this, we carried out a targeted 16S rRNA gene amplicon sequencing based on genomic DNA (gDNA), as well as complimentary DNA (cDNA). gDNA was considered as representative for the whole present bacterial community, whereas cDNA represented the metabolically active part of this community on the premise that only active bacterial cells harbored intact ribosomal RNA molecules—a substrate in the applied metatranscriptomic procedure. Nucleic acids were extracted from bacterial communities residing in nitrophilous, nitrogen-tolerant, and nitrogen-sensitive lichen species, from two distinct habitats at King George Island, maritime Antarctica: the nitrogen abundant area surrounding the Point Thomas penguin rookery and the nitrogen-depleted area near Jardine Peak. Using these complementary types of data (cDNA and gDNA) allows to confer a more holistic picture of the impact that the polar environment poses on the bacterial community residing within lichens, as well as the microbe–host interactions taking place in one of the world’s harshest, yet most intriguing ecosystem.

## Materials and methods

### Lichen sampling scheme

**Figure 1.**
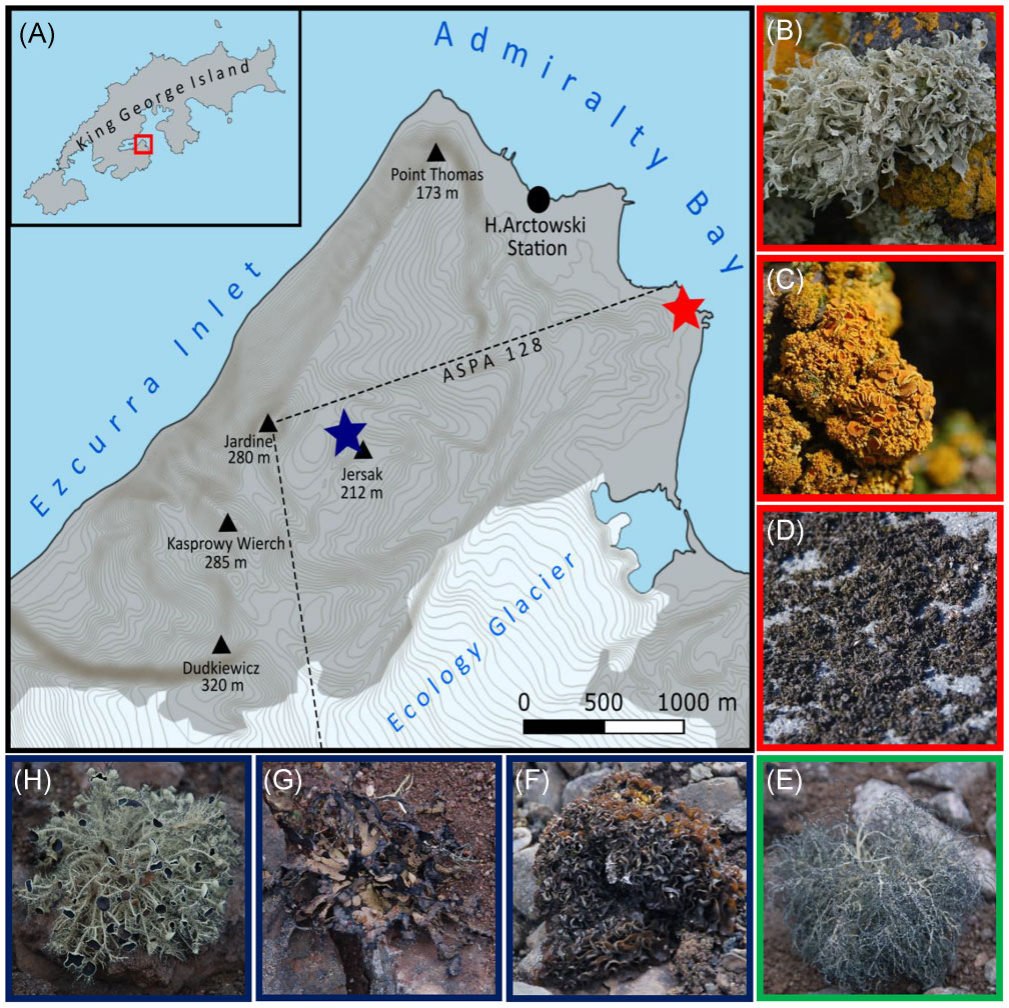
Sampling sites and materials. **(A)** map of the sampling site; red star—Point Thomas Penguin Rookery area, blue star—sampling site within the Jarden Peak area. Photographs of lichen specimens: **(B)***Ramalina terebrata;***(C)***Gondwania regalis;***(D)***Turgidosculum complicatulum;***(E)***Usnea antarctica;***(F)***Leptogium puberulum;***(G)***Himantormia lugubris;***(H)***Usnea aurantiaca-atra*.

Samples were obtained during the 43rd expedition to the Polish Antarctic Station “Arctowski,” from ice-free areas at the western shore of Admiralty Bay (King George Island, Antarctica), as well as the barren terrains that border the southern shore of the Ezcurra Inlet (Table 1, Fig. 1A). Lichen specimens were collected from areas with varying nutrient inputs (Olech 2004). Sampling was done within the span of 8 h in March of 2019. Samples from the nitrophilous (ornitocoprophilous) lichen communities included the species: *Ramalina terebrata* Hook f. and Taylor (Fig. 1B), *Gondwania regalis* (*Caloplaca regalis*) (Vain.) Søchting, Frödén and Arup (Fig. 1C), and *Turgidosculum complicatulum* (*Mastodia tesselata*) (Nyl.) J. Kohlm. and E. Kohlm. (Fig. 1D) procured from the nutrient-rich site of the Point Thomas penguin rookery. Samples from the nitrophobic lichen communities included the species: *Leptogium puberulum* Hue (Fig. 1F), *Himantormia lugubris* (Hue) I. M. Lamb (Fig. 1G), *Usnea aurantiaco-atra* (Jacq.) (Fig. 1H) and were collected at the nutrient-lacking site near the Jardine Peak (Southern shore of the Ezcurra Inlet). Thalli samples of the nitrogen-tolerant lichen species *Usnea antarctica* Du Rietz (Fig. 1E) were obtained from both sites. Three specimens of a particular species were collected per site with the use of sterile tweezers and scissors. Thalli samples were immediately submerged into 30 ml of StayRNA™ solution (A&A Biotechnology) in 50 ml conical tubes and stored overnight in 4°C, as suggested in the user’s manual. On the following day, the samples where rid of the remaining solution and stored in −20°C until further analysis. Taxonomic identification of the lichen specimens was carried out by Maria A. Olech.

**Table 1.** Sampling sites’ characteristics. GR—*Gondwania regalis*; RT—*Ramalina terebrata*; TC—*Turgidosculum complicatulum*; UP—*Usnea antarctica*, penguin rookery; HL—*Himantormia lugubris*; UA—*Usnea aurantiaco-atra*; LJ—*Leptogium puberulum*; and UJ—*Usnea antarctica*, Jardine Peak.

Sample	Site description	Coordinates
**GR**	Point Thomas penguin rookery; flat planes covered with decaying penguin excreta and weathered basaltic rocks of varying height occupied by a nitrophilous/ornitocoprophilous and nitrogen tolerant lichen community	62.16336 S
		58.46022 W
**RT**		62.16978 S
		58.45986 W
**TC**		62.15311 S
		58.46311 W
**UP**		62.16328 S
		58.45903 W
**HL**	Jardine Peak area; mostly loose rocks and gravel; nitrophobic lichen communities on neighboring rock walls, with the addition of nitrogen tolerant lichens growing in the vicinity	62.166 S
		58.49272 W
**UA**		62.16733 S
		58.49142 W
**LJ**		62.15567 S
58.48528 W		
		
**UJ**		62.16594 S
		58.47036 W

### Nucleic acid isolation

DNA and RNA were coextracted from the StayRNA™ preserved lichen thalli samples. In brief, 0.2 g of the sampled thallus was ground with a sterile pestle and mortar, with the addition of liquid nitrogen and sterile sharp, garnet sand (Lysing Matrix A, MP Biomedicals). The ground sample was divided into roughly equal aliquots for DNA and RNA isolations.

RNA isolation was carried out under a UV cabinet. All surfaces and equipment were cleaned with the labZap™ solution (A&A Biotechnology). Approximately, 0.1 g of the ground sample was mixed with 1 ml of TRI REAGENT^©^ (Molecular Research Center, Inc.) and incubated for 5 min/RT, after which 0.2 ml of chloroform was added and incubated for 3 min/RT. The samples were centrifuged at 12 000 × *g*/15 min/4°C and the RNA-containing aqueous phase transferred into a new microcentrifuge tube. This step was carried out twice. Next, 3 vol of 96% EtOH and 1/10th vol of 3 M sodium acetate (pH 5.2) solution were added, and the samples were left overnight in −80°C. The following day, the samples were vortexed and left to sit for 10 min/RT, after which they were centrifuged at 7500 × *g*/5 min/4°C. The RNA was visible as a small white pellet. The pellet had been washed thrice with 75% EtOH and centrifuged at 7500 × *g*/5 min/4°C. The samples were air-dried in RT. The pellet was resuspended in 50 µl DEPC-treated, nuclease-free water (A&A Biotechnology) and incubated at 4°C to solubilize the RNA. After vortexing the samples, the concentration and purity were checked on a NanoPhotometer^®^ NP80 (Implen). The quality of the isolated RNA was further improved by the use of the Clean-up RNA Concentrator kit (A&A Biotechnology), which included a DNA removal step with the use of a DNase, and checked once again on a NanoPhotometer^®^ NP80 (Implen). To ensure that the RNA was completely deprived of DNA, a PCR reaction was set up, with the RNA samples serving as a template and *Escherichia coli* dh5α gDNA serving as a positive control. PCR reaction had been conducted using the high specificity ready-to-use mix PCR Mix Plus (A&A Biotechnology) in a final volume of 25 μl per reaction, according to the user’s manual. The primers used were gene-specific primers: 16S_V3-F and 16S_V4-R, which cover positions 341–357F and 785–805R, respectively, according to *E. coli* 16S rRNA gene reference sequence (Klindworth et al. 2013). PCR product presence was checked on an agarose gel electrophoresis. DNase-treated and -cleaned RNA samples were stored until further analysis in −80°C. The reverse transcription was carried out with the use of QuantiNova Reverse Transcription Kit (Qiagen) containing random hexamer primers according to the manufacturer’s instructions. The obtained 24 cDNA samples were stored in −20°C until further analysis.

Total DNA was isolated using the CTAB method, according to the protocol by Wilson (2001) featured in *Current protocols in Molecular Biology*, after which a concentration and purity check was conducted on a NanoPhotometer^®^ NP80 (Implen). The samples were cleaned up with a Clean-up Concentrator kit (A&A Biotechnology), according to the user manual and followed-up with another concentration and purity check. The obtained 24 gDNA samples were stored in −20°C until further analysis.

### 16S rRNA gene targeted amplicon sequencing of cDNA and gDNA

For the Illumina 16S rRNA-targeted amplicon sequencing, a PCR reaction had been conducted in triplicate using the WALK polymerase (*Pwo* polymerase with proof-reading activity) (A&A Bioetchnology), in a final volume of 25 μl per reaction, according to the user’s manual. The primers used were gene-specific primers: 16S_V3-F and 16S_V4-R, which cover positions 341–357F and 785–805R, respectively, according to *E. coli* 16S rRNA gene reference sequence (Klindworth et al. 2013). The primers contained the Illumina Nextera XT (Illumina, San Diego, USA) overhang adapter nucleotide sequences, both added to the primer pair sequences for compatibility with Illumina index and sequencing adapters. The amplification conditions for both sets of primers were as follows: initial denaturation at 95°C/3 min, followed by 30 cycles of denaturation at 95°C/30 s, annealing at 58°C/30 s, and elongation 72°C/30 s, with the final elongation at 72°C/5 min. The obtained products were pooled in equimolar ratio within a particular lichen species (and site for generalist lichens—see Table 1) and indexed using the aforementioned Nextera XT barcodes (Illumina). Amplicon libraries were pooled and sequenced on the Illumina MiSeq instrument (Illumina) at the DNA Sequencing and Synthesis Facility (Institute of Biochemistry and Biophysics, Polish Academy of Sciences), with the sequencing conducted in paired-end mode (2 × 300 bp) with the use of a v3 (600 cycles) chemistry cartridge, which allowed generation of long paired reads fully covering 16S V3–V4 amplicons. A total of 16 amplicon sequence sets were obtained, eight based on gDNA and eight based on cDNA.

### Data analysis

Raw sequencing data were cleaned, aligned, and classified automatically by the EzBioCloud platform using the PKSSU4.0 database (Yoon et al. 2017). Chimeric, low quality, and nontarget (chloroplast, mitochondrial, and archaeal) amplicons were automatically discarded. The operational taxonomic unit (OTU) was defined as a group of sequences that exhibit greater than 97% similarity to each other. Illumina reads were deposited in the NCBI Sequence Read Archive (SRA) as BioProject PRJNA873246. All results were compiled using Excel 2016 (MS Office) for Windows. Correlations between family-rank sequence abundances derived from gDNA and cDNA data were calculated using Pearson’s correlation coefficient. Principal component analysis (PCA) of lichen-associated bacterial communities based on family-rank group abundances was performed using the singular value decomposition method. Data visualization and statistical analysis have been performed using the R software (R v.4.0.2) and the following packages: ggplot2, pheatmap, ggvenn, fmsb, Hmisc, ggpubr, corrplot, and autoplot (R Core Team 2013).

## Results

**Figure 2.**
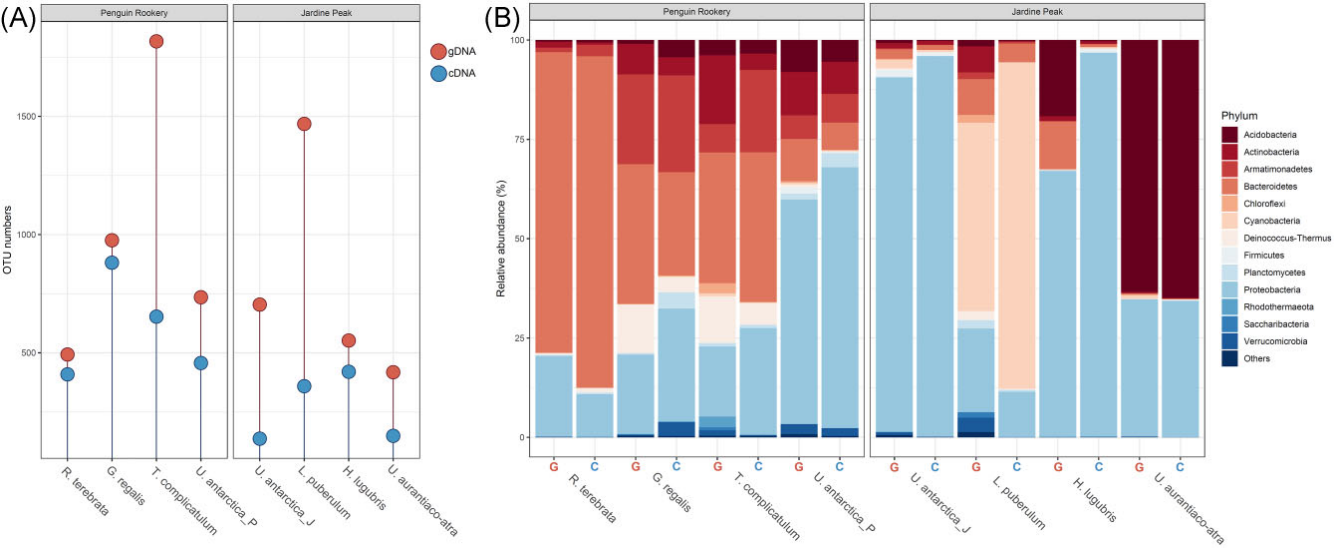
Sequence abundance of lichen-associated bacterial communities based on gDNA and cDNA analysis. **(A)** OTU numbers based on 97% sequence similarity. **(B)** Relative abundance of sequences identified on a phylum level. g—gDNA, c—cDNA, _P—Point Thomas penguin rookery area, and _J—Jardine Peak area.

Target reads obtained in the gDNA analysis ranged between 7506 and 79 834, while those obtained in the cDNA analysis between 3750 and 40 484. Bacterial OTU numbers based on gDNA analysis ranged from 418 to 1817 (av. 895.4, sd. 500.6), based on cDNA the OTU numbers were in the range of 137–881 (av. 433.1, sd. 246.2). Lowest discrepancies between gDNA and cDNA derived OTU numbers were noted for *R. terebrata* and *G. regalis* (84 and 95, respectively), while the highest in *T. complicatulum* (1164 OTUs) and *L. puberulum* (1109 OTUs) (Fig. 2A).

Phylum-rank bacterial community composition followed mostly a lichen species-specific pattern (Fig. 2B). gDNA and cDNA data indicate that the species *U. antarctica* was dominated by members of the phylum Proteobacteria: Jardine Peak area sampled *U. antarctica* specimens displayed very high Proteobacteria contribution (gDNA—86%, cDNA—96%), while the Penguin rookery specimens showed lower abundances of Proteobacteria (gDNA—56.5%, cDNA—65.6%) and high relative abundances of Actinobacteria (gDNA—10.8%, cDNA—8%), Bacteroidetes (gDNA—10.6%, cDNA—6.9%), Acidobacteria (gDNA—8.1%, cDNA—5.5%), and Armatimonadetes (gDNA—5.9%, cDNA—7.22%). The species from nitrophilic lichen communities were very abundant in Bacteroidetes bacteria: *R. terebrata* (gDNA—75.7%, cDNA—83.4%), *G. regalis* (gDNA—35%, cDNA—26%), *T. complicatulum* (gDNA—33%, cDNA—37.6%). *Gondwania regalis* also contained high amounts of Armatimonadetes (gDNA—22.6%, cDNA—24%), Proteobacteria (gDNA—20%, cDNA—28.4%), Actinobacteria (gDNA—7.7%, cDNA—4.5%), and Deinococcus-Thermus members (gDNA—12.2%, cDNA—3.8%). *Turgidosculum complicatulum* community displayed a similar structure. *Usnea aurantiaco*-*atra* community displayed unusually high contribution of Acidobacteria (gDNA—63%, cDNA—65%), followed by Proteobacteria (gDNA—34.5%, cDNA—34.3%). *Himantormia lugubris* community was dominated by Proteobacteria (gDNA—67%, cDNA—96.6%), while considerable amounts of Acidobacteria (19.22%) and Bacteroidetes (12%) were present only in the gDNA samples. *Leptogium puberulum* community composition was largely dominated by Cyanobacteria (gDNA—47.4%, cDNA—82.15%), followed by Proteobacteria (gDNA—21%, cDNA—11.4%).

**Figure 3.**
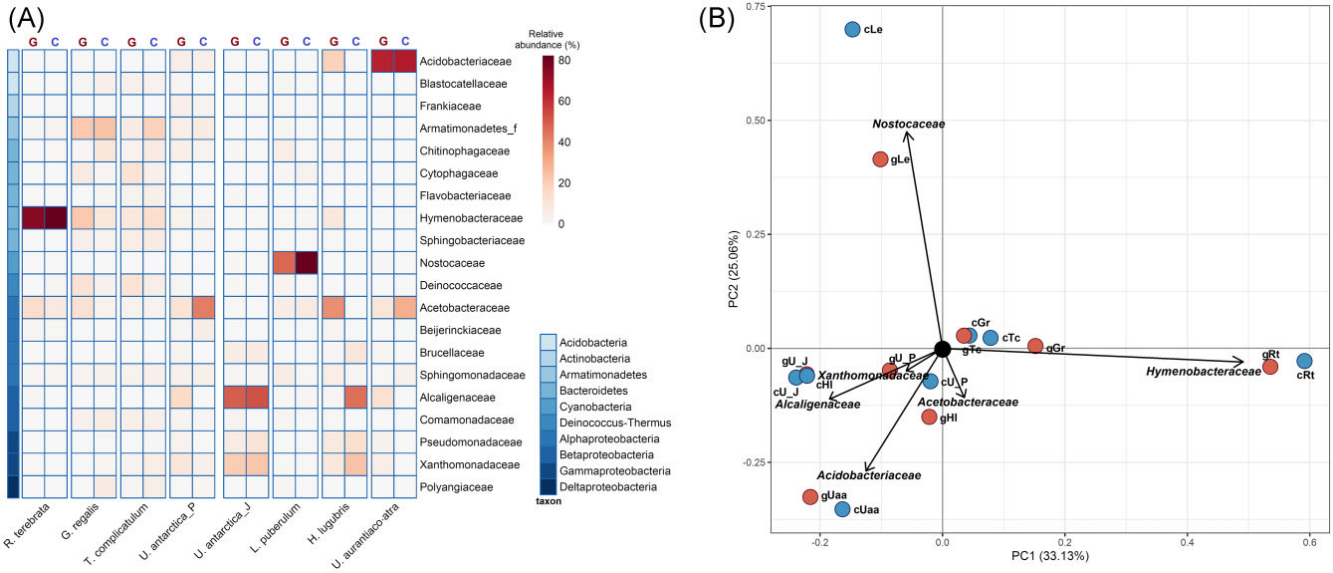
Comparison between lichen-associated bacterial communities on a family-rank taxonomic level. **(A)** Relative abundance of sequences identified on a family level; G—gDNA, C—cDNA. **(B)** PCA of lichen-associated bacterial communities based on family-rank group abundances; gDNA (red dots), cDNA (blue dots). Gr—*G. regalis*, Rt—*R. terebrata*, Tc—*T. complicatulum*, Hl—*H. lugubris*, Uaa—*U. aurantiaco*-*atra*, Le—*L. puberulum*, U_P—*U. antarctica* at penguin rookery, and U_J—*U. antarctica* at Jardine Peak.

Relative abundances of sequences identified on a family-rank level displayed severe differences between lichen species, but also between gDNA and cDNA-derived data (Fig. 3A). *Gondwania regalis* bacterial community based on gDNA sequencing consisted mostly of *Hymenobacteraceae* (22.2%), an Armatimonadates family (22.1%), *Acetobacteraceae* (12.5%), and *Deinococcaceae* (12.2%) family members. Data derived from cDNA sequencing showed a community with a comparable relative abundance of the Armatimonadates family (23.9%) while also being rich in *Chitinophagaceae* members (10,2%). Similarly, *T. complicatulum* community consisted of many families in moderate abundances, most notably the *Deinococcaceae* (gDNA—11.6%, cDNA—5.4%), *Cytophagaceae* (gDNA—11.2%, cDNA—4.2%), and*Hymenobacteraceae* (gDNA—8.5%, cDNA—14.2%), an Armatimonadates family (gDNA—6.9%, cDNA—18.8%) and *Sphingobacteriaceae* (gDNA—6%, cDNA—7%). *Ramalina terebrata* community was dominated by the *Hymenobacteraceae* family members, both in the gDNA (75.1%) and cDNA (82%) analysis, while also being rich in *Acetobacteraceae* (gDNA—15%, cDNA—7.5%). *Usnea aurantiaco*-*atra* harbored mainly members of the family *Acidobacteriaceae* (gDNA—63.3%, cDNA—64.9%), but *Acetobacteraceae* were also abundant (gDNA—10.3%, cDNA—30%). *Alcaligenaceae* featured only in gDNA-based results for this species community (13.1%). *Himantormia lugubris* differed in family rank sequence abundance between gDNA and cDNA-derived data. For the cDNA, the highest relative abundances were obtained for the *Acetobacteraceae* (37.9%), *Acidobacteriaceae* (19%), *Hymenobacteraceae* (8.8%), and *Xanthomonadaceae* (8.7%), while for the cDNA data the *Alcaligenaceae* (45.4%), *Xanthomonadaceae* (23.4%), and *Pseudomonadaceae* (13.9%) were the most abundant families. *Leptogium puberulum* community consisted mostly of the cyanobacterial family *Nostocaceae* (gDNA—47.3%, cDNA—82.1%), with *Acetobacteraceae* being also largely present (gDNA—5.4%, cDNA—7.1%). Large discrepancies were noted for the generalist (eurytopic) lichen species *U. antarctica*, mostly between sites, but also between DNA templates. *Usnea antarctica* community from the Penguin rookery harbored, based on gDNA analysis, moderate amounts of sequences belonging to several families, most notably *Alcaligenaceae* (16.1%), *Acetobacteraceae* (11.2%), and *Xanthomonadaceae* (10%), while based on the cDNA sequencing results *Acetobacteraceae* dominated the community (42.2%). *Usnea antarctica* community from the Jardine Peak area showed great similarity between gDNA- and cDNA-derived data and consisted mostly of *Alcaligenaceae* (gDNA—49.4%, cDNA—52.4%), *Xanthomonadaceae* (gDNA—20.9%; cDNA—22.7%),*Pseudomonadaceae* (gDNA—6.3%, cDNA—10.9%), and *Brucellaceae* (gDNA—5.6%, cDNA—6.9%).

The PCA clustered the different bacterial communities according to relative abundances of family-rank sequences (Fig. 3B). *Ramalina terebrata* community showed the greatest dissimilarity to the rest of the lichen-associated community with the *Hymenobacteraceae* relative abundance being the most significant determining factor. The *L. puberulum* community was also noticeably distant, with the *Nostocaceae* relative abundance significantly influencing the clustering outcome. *Acidobacteriaceae* relative abundance defined a cluster consisting of *U. aurantiaco-atra* community (gDNA and cDNA results). The relative abundance of *Acetobacteraceae* was significant for the *H. lugubris* gDNA community placement, whereas the relative abundance of *Alcaligenaceae* and *Xanthomonadaceae* defined the *U. antarctica* community (gDNA and cDNA) at the Penguin rookery, as well as the cDNA-derived *H. lugubris* community. A somewhat coherent group was formed by the Penguin rookery lichen-associated communities of *G. regalis* and *T. complicatulum*.

**Figure 4.**
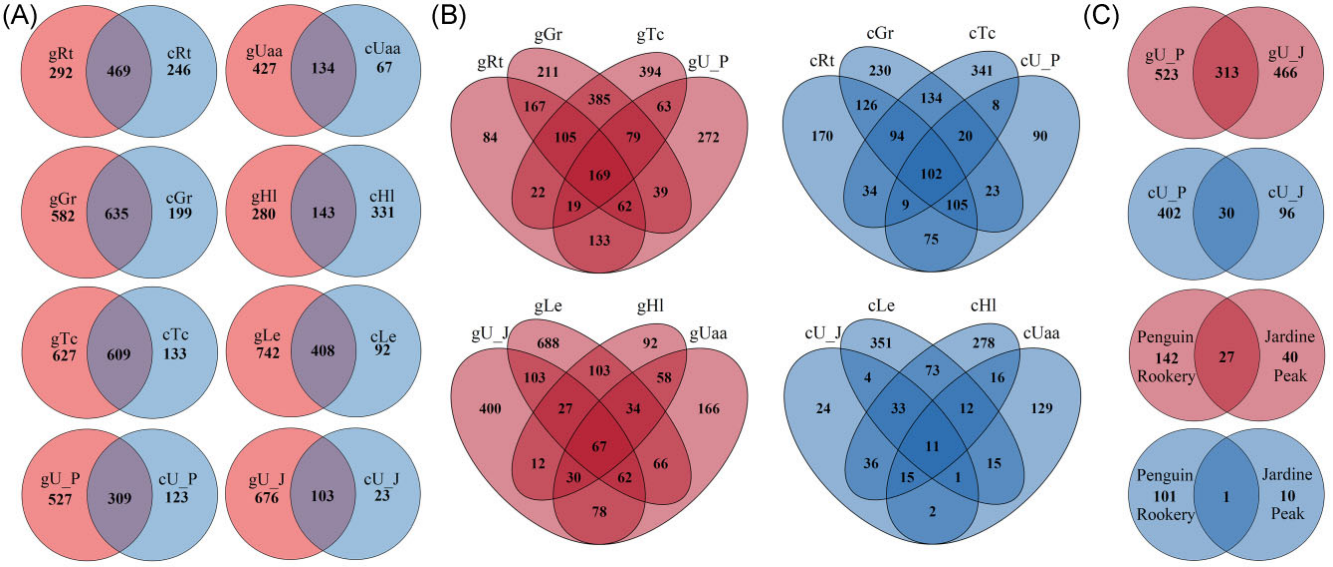
Venn diagrams displaying shared and individual OTUs (97%) derived from gDNA (red) and cDNA (blue). **(A)** Shared and individual OTUs derived from gDNA and cDNA within one sample group. **(B)** Shared and individual OTUs within four sample groups compared according to DNA template and habitat: penguin rooker—upper row, Jardine Peak—lower row. **(C)** Shared and individual OTUs of *U. antarctica* communities and core OTUs for penguin rookery and Jardine Peak lichens. Gr—*G. regalis*, Rt—*R. terebrata*, Tc—*T. complicatulum*, Hl—*H. lugubris*, Uaa—*U. aurantiaco*-*atra*, Le—*L. puberulum*, U_P—*U. antarctica* at penguin rookery, U_J—*U. antarctica* at Jardine Peak, penguin rookery—core OTUs of penguin rookery lichen communities, and Jardine Peak—core OTUs of Jardine Peak lichen communities.

Similarities between DNA templates based on shared OTU numbers showed several phenomena (Fig. 4A). Bacterial communities of the nitrophilic species *G. regalis, R. terebrata*, and *T. complicatulum* showed much similarity between DNA templates (635, 469, and 609 of shared OTUs, respectively), while the communities associated with the nitrophobic species *H. lugubris, U. aurantiaco-atra*, and *L. puberulum* showed relatively low similarity between templates (143, 134, and 408 shared OTUs between templates, respectively). There were site-specific differences in shared OTU numbers between templates for the generalist lichen communities. The *U. antarctica* community at the Penguin rookery shared 309 OTUs between the gDNA and cDNA template whereas the Jardine Peak *U. antarctica* community shared only 103 OTUs. The number of OTUs shared between the four lichen species growing at the penguin rookery (gDNA—169; cDNA—102) were substantially higher than those shared by the lichen species growing in the Jardine Peak area (gDNA—67; cDNA—11) (Fig. 4B). Based on the gDNA analysis, *U. antarctica* bacterial communities shared 313 OTUs between sites, yet only 30 based on cDNA sequences. Only 27 OTUs were shared between the core communities of the penguin rookery and Jardine Peak area for the gDNA template and one for the cDNA template (Fig. 4C).

**Figure 5.**
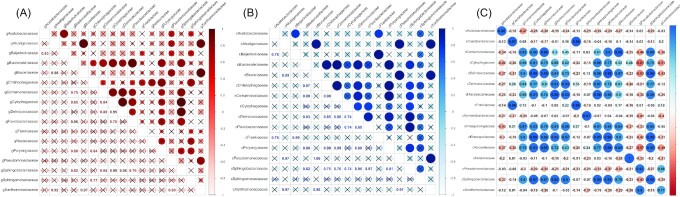
Correlations between family-rank sequence abundances displayed as Pearson’s correlation coefficient values. **(A)** Correlogram of family-rank sequence abundances derived from gDNA data (significance level at *P*< .05, insignificant correlation are crossed out). **(B)** Correlogram of family-rank sequence abundances derived from cDNA data (significance level at *P*< .05, insignificant correlation are crossed out). **(C)** Family-rank sequence abundances correlogram derived from gDNA data and cDNA.

Within the gDNA-derived data the relative abundance of the family *Sphingobacteriaceae* (phylum Bacteroidetes) and *Blastocatellaceae* (phylum Acidobacteria) displayed the highest numbers of significant (*P* ≤ .05) correlations with relative abundances of other families (Fig. 5A). Relative abundance of *Sphingobacteriaceae* displayed positive significant correlations with the relative abundances of the following families: *Blastocatellaceae* (ρ = 0.82, *P* = .013), *Comamonadaceae* (ρ = 0.84, *P* = .009), *Cytophagaceae* (ρ = 0.99, *P* = 1.04 × 10^−6^), *Deinococcaceae* (ρ = 0.96, *P* = .00018), and *Flavobacteriaceae* (ρ = 0.76, *P* = .03). The relative abundance of the *Blastocatellaceae* had the highest correlation values with the relative abundances of *Flavobacteriaceae* (ρ = 0.96, *P* = .0001) and *Cytophagaceae* (ρ = 0.85, *P* = .008). The relative abundance of the *Xanthomonadaceae* displayed significant correlations with the relative abundances of: *Alcaligenaceae* (ρ = 0.92, *P* = .001), *Brucellaceae* (ρ = 0.97, *P* = .00008), and *Pseudomonadaceae* (ρ = 0.80, *P* = .02). The relative abundance of *Comamonadaceae* had positive significant correlations with the *Deinococcaceae* (ρ = 0.88, *P* = .004) and *Cytophagaceae* (ρ = 0.84, *P* = .008).

Based on the cDNA analysis (Fig. 5B), the relative abundance of the family *Sphingobacteriaceae* displayed the highest number of positive significant correlations with the relative abundances of other families, most notably with: the *Blastocatellaceae* (ρ = 0.82, *P* = .01), the *Chitinophagaceae* (ρ = 0.75, *P* = .03), the *Comamonadaceae* (ρ = 0.76, *P* = .028), the *Cytophagaceae* (ρ = 0.74, *P* = .035), the *Deinococcaceae* (ρ = 0.95, *P* = .0003), the *Flavobacteriaceae* (ρ = 0.97, *P* = .0001), and the *Polyangiaceae* (ρ = 0.80, *P* = .015). Relative abundance of the *Blastocatellaceae* displayed significant correlations with the abundances of the following families: *Chitinophagaceae* (ρ = 0.97, *P* = .0001), *Comamonadaceae* (ρ = 0.99, *P* = 3.38 × 10^−6^), *Deinococcaceae* (ρ = 0.85, *P* = .007), and *Polyangiaceae* (ρ = 0.99, *P* = 4.05 × 10^−7^). The *Chitinophagaceae* also displayed positive correlations with the *Comamonadaceae* (ρ = 0.98, *P* = 9.22 × 10^−6^) and *Deinococcaceae* (ρ = 0.85, *P* = .006). The relative abundance of the *Xanthomonadaceae* family correlated positively with the abundances of *Alcaligenaceae* (ρ = 0.97, *P* = 6.19 × 10^−5^), *Brucellaceae* (ρ = 0.95, *P* = .0002), and *Pseudomonadaceae* (ρ = 0.97, *P* = 4,6 × 10^−5^).

A total of 16 lichen-associated bacterial families displayed significant (*P* ≤ .05) positive correlations within their respective relative abundances derived from gDNA and cDNA data analysis (Fig. 5C), including those that featured in considerable amounts (≥ 4%) in any of the examined samples. Highest coefficient values were noted for: *Nostocaceae* (ρ = 1, *P* = 1.75 × 10^−11^), *Frankiaceae* (ρ = 0.98, *P* = 1.45 × 10^−5^), *Hymenobacteraceae* (ρ = 0.97, *P* = 4.89 × 10^−5^), *Sphingobacteriaceae* (ρ = 0.96, *P* = .00015), *Acidobacteriaceae* (ρ = 0.96, *P* = .0002), *Deinococcaceae* (ρ = 0.95, *P* = .0003), *Flavobacteriaceae* (ρ = 0.93, *P* = .0009), *Pseudomonadaceae* (ρ = 0.92, *P* = .001), *Comamonadaceae* (ρ = 0.85, *P* = .008), *Cytophagaceae* (ρ = 0.81, *P* = .014), and *Xanthomonadaceae* (ρ = 0.77, *P* = .025).

## Discussion

Despite the extensive knowledge about the input of lichens into the Earth’s ecosystem, there is still much to learn about these pioneering metaorganisms, especially regarding the diversity of the taxa partaking in this multispecies symbiosis in Antarctica (Grimm et al. 2021). In this study, we showed how amending metagenomic information with metatranscriptomic data allows for a more detailed description of the heterotrophic bacterial component that actively contributes to the lichen symbiosis. This additional information is especially important when comparing lichen bacteriomes in different trophic surroundings, as most profound changes in diversity can be seen through the activity of specialized phylogenetic and/or functional fractions, something that can only be studied through transcriptomic analyses (Cernava et al. 2019).

The Point Thomas penguin rookery is considered an Antarctic Specially Protected Area due to its unique biodiversity (Chwedorzewska and Korczak 2010). Organisms found at this site either thrive on, or tolerate the high concentrations of ammonia fumes that come off the decaying guano, making it a major environmental factor exerting pressure on this biocenosis (which is also influenced by marine factors, as it is located at the shore of Admiralty Bay) (Olech 2004, Myrcha et al. 2013, Grzesiak et al. 2020). Bacterial communities of lichens procured from the penguin rookery displayed a high degree of similarity (as shown in the PCA outcome), with the exception of the *R. terebrata* communities. Nevertheless, the amount of OTUs shared between all four communities was relatively high, particularly between their active fractions. This suggests that a consortium of bacteria adapted to the prevalent conditions has been established there. A vital part of this group of bacteria belonged to the Bacteroidetes phylum, which have been found to dominate, or massively contribute, to the bacterial communities of the Point Thomas penguin rookery lichens. This phylum’s occurrence has also been noted for the strictly marine lichens of the Brittany coast (West et al. 2018). Authors of this paper discovered the highest Bacteroides abundance in marine lichens, hinting that seawater influences may be the cause of this phenomenon. However, other lichens investigated by West et al. (2018) (inland cyanolichens and maritime, nitrophilous lichens of the genus *Xanthoria*), were also considerably rich in Bacteroidetes. Given our findings, a more plausible explanation can be given, that the Bacteroides bacteria tend to occupy lichens experiencing a relatively high abundance of nitrogen, be it of endogenic (cyanobiont N_2_ fixation) or exogenic nature. Additionally, cDNA data revealed that Bacteroidetes were transcriptomically active only in the penguin rookery lichens and in the examined cyanolichen *L. puberulum*. The findings regarding the *U. antarctica* bacterial community further corroborate this thesis, with specimens growing at the rookery housing a substantial amount of Bacteroidetes members, while specimens of the Jardine Peak area carrying only minuscule amounts. The extent of Bacteroidetes dominance, seen here in *R. terebrata*, has also been observed in the lichen *Umbilicaria decussata* (a nitrogen-tolerant species) procured from the shore of Lützow-Holm Bay, coastal Queen Maud Land, continental Antarctica, where several large penguin and petrel breeding colonies are located (He et al. 2022). The majority of the *R. terebrata* bacteriome sequences were assigned to the family *Hymenobacteraceae*, which were also present in the three other penguin rookery lichen species. Information on the involvement of *Hymenobacteraceae* members in the lichen symbiosis is limited, however, a genome study on a few lichen-associated *Hymenobacter* spp. isolates revealed their potential resistance to UV radiation, as well as the ability to decompose complex polysaccharides (Ahn et al. 2016, Oh et al. 2016, Ghimire et al. 2020). *Ramalina terebrata* grows on vertical or greatly inclined rock walls, which are periodically under intense sunlight irradiation (Olech 2004). The species is also known for producing usnic acid and ramalin as UV protectant and antioxidant, respectively (Quilhot et al. 1996, Paudel et al. 2011). These, and a few other compounds produced by *R. terebrata* were confirmed as having an antimicrobial effect (Paudel et al. 2010). Consequently, the combination of these abiotic and biotic influences may have led to the development of a bacterial community, i.e. low in diversity, yet containing many multiresistant members, making them almost the exclusive inhabitants of the *R. terebrata* thalli. Interestingly, *U. decussata* is also known as a gyrophoric acid producer, which displays potent antimicrobial activity (Olech 2004, Ranković et al. 2008). The abundance of *Hymenobacteraceae* did not correlate with the abundance of any other bacterial family that was considerably contributing to the Antarctic lichens’ communities in this study, yet their abundance based on gDNA positively correlated with that based on cDNA data. This shows their potential to form monoculture-like assemblages in the lichen thalli, and that members of this family are active in lichen-associated communities whenever they are present. Besides Bacteroidetes, the two other nitrophilic lichen species: *T. complicatulum* and *G. regalis* also harbored considerable amounts of bacteria identified as Armatimonadetes by the database used. Available information points toward Armatimonadetes preference for nitrophilic, sea coast-dwelling chlorolichens (West et al. 2018, He et al. 2022). Members of this group are rarely cultivated, so the knowledge on their environmental role and features are scarce, however, fairly recently, a new member of this taxon was isolated from Antarctic soil—*Abditibacterium utsteinense* (Tahon et al. 2018). Coincidentally, one of the most abundant OTUs shared among the communities associated with the lichens of the Point Thomas penguin rookery examined in this study was identified as the genus *Abditibacterium* (data not shown). Physiological and genomic investigations revealed that *A. utsteinense* is well-adapted to coping with environmental stresses present in Antarctica: oxidative and radiation stress, temperature fluctuations but also to toxic compounds (Tahon et al. 2018). The strain had a very narrow pH growth range, hinting that this bacterium is adapted to a stable environment, not unlike a lichen thallus, for which the pH homeostasis is crucial for survival. Furthermore, it contained the genetic basis for a versatile nitrogen metabolism, including ammonia transporters, as well as the ability to sequester ammonia by ʟ-glutamine synthesis, which is a way to reduce NH_4_^+^ concentration, thus avoiding its toxic effects—a major issue for organisms experiencing the aforementioned ammonia shadow effect (Dahlman et al. 2003, Crittenden et al. 2015).

The Jardine Peak area constitutes a highland plateau where on rocky substrates, such as exposed rock faces, ridges, and stony slopes, nitrophobic lichen communities develop (Olech 2004). Lichen-associated microbiomes sampled there were dominated by Proteobacteria, however, the communities were mostly host species-specific, as indicated by the low numbers of shared OTU’s, especially those based on cDNA data. The most distinctive bacterial community among the Jardine Peak chlorolichens was found residing in *U. aurantiaco-atra*. It was composed mostly of Acidobacteria. This feature, to a greater or lesser extent, was also observed for the lichen species *Cladonia borealis* and *Ochrolechia parallela*, procured from nitrogen-deficient sites at King George Island (Antarctica) (Park et al. 2016). Additionally to the preference for nutrient-depleted habitats, these lichens have the ability to individually produce a variety of complex, bioactive, and acidic compounds (Olech 2004). Consequently, the combination of low intrathallic pH, preference for oligotrophy and inhibitory compound presence likely led to the establishment of an Acidobacteria-dominated community, due to the specific niche requirements of these bacteria (Kielak et al. 2016). Most of the Acidobacteria sequences from *U. aurantiaco-atra* were identified as belonging to the *Acidobacteriaceae* family. The abundance of this family did not correlate with the abundance of any other family, yet as with the *Hymenobacteraceae*, their abundance by gDNA-based analysis did correlate with cDNA-based abundance, hinting their presence is not influenced by interactions with other microbes, but by abiotic- and lichen-based factors, with pH likely serving as the most critical one. Some culture-based studies indicate that members of this family can grow on lichen-specific polymers like lichenan, as well as chitin and cellulose (Belova et al. 2018). *Himantormia lugubris* displays a curious case, where the structure of the active part of the bacterial community is distinctly different than that of the whole community. The latter was especially rich in *Acetobacteraceae* (Alphaproteobacteria), while the active part of this community was defined by the abundances of *Alcaligenaceae* (Betaproteobacteria) and *Xanthomonadaceae* (Gammaproteobacteria). Such discrepancies in the community structure, where a biodiverse reservoir of dormant bacteria was created, indicates that *H. lugubris* undergoes seasonal changes within intrathallic conditions (Cruaud et al. 2020). What is known of *H. lugubris* ecophysiology is that it has great tolerance for desiccation, as well as being covered with snow for extended periods of time (Choi et al. 2015). It has also been concluded that the production of inhibitory phenolic compounds ceases in *H. lugubris* during sunlight exposure (Mateos et al. 1991). The associated bacterial community may, therefore, respond to these changes by shifting the abundance of its active participants. Furthermore, the active part of the *H. lugubris* community bears a very close resemblance to the *U. antarctica* community of Jardine Peak (whole and active part). The two families whose abundance defines these communities displayed a positive correlation with each other, suggesting a symbiotic relation. However, only *Xanthomonadaceae* displayed a positive correlation between their gDNA/cDNA abundances, meaning that *Alcaligenaceae* are more dependent on their presence than *vice versa*. The abundance of *Alcaligenaceae* was noticeably higher in Jardine Peak specimens of *U. antarctica* and *H. lugubris*, suggesting these bacteria are advantageous in oligotrophic conditions. Some members of this family are endosymbiotic and nitrogen fixing (Taulé et al. 2012). Curious in this scenario is the positive correlation with the *Xanthomonadaceae*, which were recognized as major antagonists in lichen-associated communities, presumably defending the resources provided by *Alcaligenaceae* (Cernava et al. 2015). Another interesting matter was the abundance of the family *Acetobacteraceae* in both *Usnea* species and *H. lugubris*. Members of this family were active in *U. antarctica* at the penguin rookery, hinting they may help the lichen cope with excess nitrogen. Noteworthy is the fact, that ammonia vapors cause a substantial alkalization of the surrounding area, consequently challenging the pH homeostasis of the non-nitrophilic lichens (Sutton et al. 2020, do Vale Lopes et al. 2021). *Usnea antarctica* may, therefore, be this tolerant of such high nitrogen concentrations due to the acidifying action of the lichen-associated *Acetobacteraceae* (Kersters et al. 2006). In the nitrophobe *H. lugubris* this family was not considerably active at the time of sampling. However, if the melting snow accumulates alkaline substances (mainly ammonia ions) and introduces them in early summer into the thallus, the properties of the *Acetobacteraceae* might prove very beneficial for the lichen (Crittenden 1998).

*Usnea antarctica* presents a case of a lichen, i.e. tolerant of high nitrogen concentrations in its immediate environment (Olech 2004). As seen in the PCA graph, the bacterial communities of *U. antarctica* did differ between the two sites. While the amount of OTUs for both these communities was similar, the number of shared OTUs between them was less than half, with concomitant, substantial differences at the phylum level. The active part of the Jardine Peak community was less diverse than that of the penguin rookery. In the lichen *Umbillicaria rhizinata* the opposite was observed, with the coastal (and nitrogen fertilized) specimens hosting a less diverse community than the inland ones (He et al. 2022). However, similarly to our *U. antarctica* samples, the coastal/nitrogen enriched specimens harbored taxa affiliated with nitrophilic lichens (Armatimonadetes, Bacteroidetes), while the inland lichens were dominated by Proteobacteria (He et al. 2022). Thus, shifting of the bacterial community structure in nitrogen-tolerant lichens (or any wide amplitude lichens) may be the basis for their efficient adaptability to changing environmental conditions. Furthermore, the findings of Cernava et al. (2015) indicate that the diversity of lichen microbiota strongly contributes to the adaptability and flexibility of the host-lichen, thus, the greater the resident bacterial diversity, the wider the ecological amplitude of the lichen species. According to our data, the *T. complicatulum* bacterial community displayed the highest number of OTUs for the whole community (gDNA data). This lichen is known to have a bipolar distribution whilst being able to thrive in high nitrogen and high salinity habitats (Olech 2004). The *L. puberulum* community was also very diverse, and despite the lichen being endemic to Antarctica, it is very widespread in the region and can be found in a range of habitats, in both inland and coastal localities, provided there is an abundance of fresh water (Olech 2004). The third most diverse bacterial community, however, was found in *G. regalis*—an ornitocoprophile, endemic to West Antarctica, restricted to sites with considerable ammonia exposure (Olech 2004). Therefore, the diversity of the whole community alone might be species-specific and not dictate the ecological amplitude of the host lichen. However, the discrepancy between the diversity of the whole and active community can suggest the scope of the adaptive potential of the bacteriocenosis, as was indicated here for the nitrogen tolerant *U. antarctica*, which occupies a variety of Southern Hemisphere habitats and is not exclusive to Antarctica (Olech 2004).

## Conclusions

Lastly, the bacterial communities of the penguin rookery lichens were distinct from the communities of the Jardine Peak area lichens due to the small number of shared OTUs. Environmental conditions present at the penguin rookery (*super omnia* ammonia vapors) largely shaped the lichen-associated microbiome, with some lichen species-specific traits, such as antimetabolite production, also influencing its structure. At the nutrient-deficient site of the Jardine Peak the lichen-dwelling microbiocenoses were distinct for each lichen species. The bacterial community of *U. antarctica* (found at both sites) displayed a high degree of flexibility. At the penguin rookery it resembled the communities found in nitrophilous lichens, while at the Jardine Peak area it harbored some bacterial groups found in nitrophobic lichens. This trait may, therefore, be a key component in the adaptability of *Usnea* and other lichen species. Given that all of the sampled lichen species had an active and a dormant fraction of its resident bacterial community, it can be concluded that the latter represents a reservoir of latent, beneficial traits that once activated can widen the ecological amplitude of the lichen holobiont, thus expanding its habitat range, even to a worldwide extent.

## Data Availability

Illumina reads were deposited in the NCBI Sequence Read Archive (SRA) as BioProject PRJNA873246.
